# Distribution of the p66Shc Adaptor Protein Among Mitochondrial and Mitochondria—Associated Membranes Fractions in Normal and Oxidative Stress Conditions

**DOI:** 10.3390/ijms252312835

**Published:** 2024-11-29

**Authors:** Magdalena Lebiedzinska-Arciszewska, Barbara Pakula, Massimo Bonora, Sonia Missiroli, Yaiza Potes, Patrycja Jakubek-Olszewska, Ines C. M. Simoes, Paolo Pinton, Mariusz R. Wieckowski

**Affiliations:** 1 Laboratory of Mitochondrial Biology and Metabolism, Nencki Institute of Experimental Biology of Polish Academy of Sciences, 02-093 Warsaw, Poland; b.pakula@nencki.edu.pl (B.P.); potesyaiza@uniovi.es (Y.P.); p.jakubek@nencki.edu.pl (P.J.-O.); inescmsimoes@gmail.com (I.C.M.S.); m.wieckowski@nencki.edu.pl (M.R.W.); 2 Department of Medical Sciences, Section of Experimental Medicine, Laboratory for Technologies of Advanced Therapies (LTTA), University of Ferrara, 44121 Ferrara, Italy; bnrmsm1@unife.it (M.B.); sonia.missiroli@unife.it (S.M.); paolo.pinton@unife.it (P.P.); 3 Department of Morphology and Cell Biology, Faculty of Medicine, University of Oviedo, 33006 Oviedo, Spain

**Keywords:** oxidative stress, fractionation, mitochondria, mitochondria—ER contact sites

## Abstract

p66Shc is an adaptor protein and one of the cellular fate regulators since it modulates mitogenic signaling pathways, mitochondrial function, and reactive oxygen species (ROS) production. p66Shc is localized mostly in the cytosol and endoplasmic reticulum (ER); however, under oxidative stress, p66Shc is post-translationally modified and relocates to mitochondria. p66Shc was found in the intermembrane space, where it interacts with cytochrome c, contributing to the hydrogen peroxide generation by the mitochondrial respiratory chain. Our previous studies suggested that p66Shc is localized also in mitochondria-associated membranes (MAM). MAM fraction consists of mitochondria and mostly ER membranes. Contact sites between ER and mitochondria host proteins involved in multiple processes including calcium homeostasis, apoptosis, and autophagy regulation. Thus, p66Shc in MAM could participate in processes related to cell fate determination. Due to reports on various and conditional p66Shc intracellular localization, in the present paper, we describe the allocation of p66Shc pools in different subcellular compartments in mouse liver tissue and HepG2 cell culture. We provide additional evidence for p66Shc localization in MAM. In the present study, we use precisely purified subcellular fraction isolated by differential centrifugation-based protocol from control mouse liver tissue and HepG2 cells and from cells treated with hydrogen peroxide to promote mitochondrial p66Shc translocation. We performed controlled digestion of crude mitochondrial fraction, in which the degradation patterns of p66Shc and MAM fraction marker proteins were comparable. Moreover, we assessed the distribution of the individual ShcA isoforms (p46Shc, p52Shc, and p66Shc) in the subcellular fractions and their contribution to the total ShcA in control mice livers and HepG2 cells. In conclusion, we showed that a substantial pool of p66Shc protein resides in MAM in control conditions and after oxidative stress induction.

## 1. Introduction

The SHC-transforming protein 1 (SHCA) subfamily of adaptor proteins comprises three isoforms, p66Shc, p52Shc, and p46Shc, which share a common, evolutionally conserved domain structure: C′-terminal SH2 domain, proline-rich collagen homology domain 1 (CH1) in the middle, and N′-terminal phosphotyrosine binding (PTB) domain. p66Shc has an additional collagen homology domain 2 (CH2) linked to the PTB domain by the cytochrome c binding (CB) region [1]. All three ShcA isoforms are predominantly localized in the cytosol and endoplasmic reticulum (ER), and are involved in the mitogenic signal transduction from receptor tyrosine kinases (RTKs) anchored in the plasma membrane to the Ras protein-related downstream signaling cascades [2]. Research revealed that p46Shc is specifically localized to the mitochondrial matrix [3], where it functions as a negative regulator of 3-ketoacylCoA thiolase (ACAA2), influencing lipid oxidation and energy metabolism [4]. p46Shc has also been observed in the nuclei of hepatocytes, particularly in proliferating cells during liver regeneration [5] and in hepatocellular carcinoma (HCC) development [6]. In HCC, both p46Shc and p52Shc show increased expression and tyrosine phosphorylation [6]. The p52Shc isoform plays a crucial role in breast cancer initiation and progression, as demonstrated by its genetic ablation significantly attenuating mammary tumor formation in rats [7]. p52Shc couples antigen and cytokine receptors to Ras activation in lymphoid and myeloid cells, while other Shc family members (including p66Shc) antagonize survival and attenuate antigen receptor signaling in lymphocytes [8]. The phosphorylation of p52Shc by c-Src is related to the increased phosphorylation of growth factor receptor–bound protein 2 (Grb2) -binding residues. This phosphorylation-dependent interaction between p52Shc and Grb2 may regulate signaling cascade activation [9]. In contrast to p46Shc and p52Shc, under oxidative stress conditions (e.g., hydrogen peroxide (H_2_O_2_), UV treatment), p66Shc becomes serine 36 (Ser36) phosphorylated by an activated protein kinase C β (PKCβ) or c-Jun N-terminal kinases (JNK) [10,11]. Ser36 phosphorylation provokes p66Shc conformational changes, which enable its transfer to mitochondria after which p66Shc accelerates reactive oxygen species (ROS) production, which triggers mitochondrial disruption and leads to cell death [11]. Consistently, the p66Shc(−/−) mice were living about 30% longer than their wild-type littermates due to increased resistance to stress and the aging process [12]. Growing evidence on the effect of oxidative stress on cells in a variety of species, including mice and humans, shows that mitochondrial dysfunction, whether primary or secondary, can further exacerbate ROS production and impair cellular energy and redox homeostasis [13,14]. The interplay between oxidative stress and mitochondrial damage creates a vicious cycle, contributing to, e.g., neuronal dysfunction and degeneration [15]. Oxidative stress, characterized by an overproduction of reactive oxygen species (ROS) which overwhelm cellular antioxidant defense systems, leads to damage of biomolecules, including DNA, lipids, and proteins [16]. Oxidative damage, particularly to mitochondria, has been implicated in pathogenetic processes [13,14,17,18]. Oxidatively damaged and aggregating cellular components may trigger autophagy [14,19]. p66Shc is therefore linked to multiple pathologies related to oxidative stress, such as diabetes, neurodegenerative diseases, cancer, and the aging process, as comprehensively reviewed in [20,21,22].

In the first reports, p66Shc was associated with the heat shock 70 kDa protein (Hsp70) in the mitochondrial matrix (MM). Oxidative stress-induced dissociation of p66Shc from Hsp70 was accompanied by increased ROS production [23]. Later, Giorgio and colleagues showed that p66Shc enters the mitochondrial intermembrane space (IMS) through a Tim/Tom complex, where it may interact with cytochrome c (Cyt c), which leads to an increase in H_2_O_2_ production, and triggers apoptosis [3,24]. In 2009, our group reported that a portion of p66Shc shifts from plasma membrane-associated membranes (PAM) to mitochondria-associated membranes (MAM) in livers of aging wild-type mice [20]. MAM contains a fraction of ER membranes that physically interact with the outer mitochondrial membrane (OMM) and comprises, depending on the experimental model and technique, from several dozen to over a thousand [25,26] proteins, involved in vital cellular processes such as calcium homeostasis regulation, lipid and phospholipid metabolism, mitochondrial dynamics, autophagy, and apoptosis. The distance between OMM and ER membranes in these contact sites is kept under tight control of tethering proteins [27]. MAMs facilitate Ca^2+^ transfer between the endoplasmic reticulum and mitochondria, influencing intracellular Ca^2+^ signaling and mitochondrial bioenergetics [28]. Type 3 inositol 1,4,5-trisphosphate receptor (IP3R3), present in ER, releases calcium ions, which pass the OMM through the voltage-dependent anion channel (VDAC) reaching mitochondrial calcium uniporter (MCU) located in the inner mitochondrial membrane (IMM). Efficient calcium ions transfer from IP3R3 through VDAC to MCU is enabled by certain tethering proteins, such as glucose-regulated protein 75 (GRP75), which maintain the proper distance between the ER and OMM ensuring that the Ca^2+^ concentration is sufficient for the MCU to sense it and transfer into MM. Ca^2+^ flux regulation is essential for activating key mitochondrial dehydrogenases, including pyruvate dehydrogenase, which are important for ATP production in mammalian cells [29,30]. Mitochondrial calcium overload, in contrast, disrupts mitochondrial physiology and activates apoptosis [31]. Pyruvate metabolism, regulated by mitochondrial Ca^2+^, is crucial for mitochondrial quality control, affecting fusion/fission dynamics and mitophagy [30]. Dysregulation of MAM function and Ca^2+^ flux can lead to altered cell death mechanisms [29,31]. Mitochondria–ER contact sites are crucial in cellular responses to oxidative stress and ER stress conditions [28,32]. Furthermore, the communication between mitochondria and ER through MAMs regulates oxidative stress and autophagy via both ROS and Ca^2+^ signaling [33]. Eukaryotic translation initiation factor 2-alpha kinase (PERK), an ER stress sensor, is enriched at MERCS [28,34]. The ER-UPR pathway, involving Ire1 and Hac1, is important for tolerating mitochondrial intermembrane space proteotoxic stress [35]. Mitochondria–ER communication also regulates mitophagy, which helps clear damaged mitochondria and prevent excessive ROS production [33]. ER chaperones and oxidoreductases regulate redox nanodomains at MERCS, influencing mitochondrial metabolism and ROS production [36]. These nanodomains, specifically H_2_O_2_ releasing, originate from mitochondrial cristae and exert positive feedback on calcium oscillations [37]. The interplay between ER stress, ROS, and calcium signaling at MERCS highlights these organelles’ complex communication and importance in cellular homeostasis and stress responses. The dysfunction of MAM was observed in pathologies such as neurodegenerative and metabolic diseases, inflammation, and cancer, as summarized in recent years by multiple literature reviews [29,30,31,38,39,40]. 

Therefore, if present in MAM, p66Shc could be in very close proximity to the mitochondria, which could be linked to the p66Shc effects on mitochondrial function. Since our report in 2009, there have been no further communications on p66Shc MAM localization, and in general, there is not much descriptive data showing the intracellular distribution of ShcA proteins, which is important for a better understanding of signal transduction in multiple models. An increase in p66Shc level in mitochondrial fraction has been observed after oxidative stress induction by factors, such as cigarette smoke, ischemia, H_2_O_2_, or phenethyl isothiocyanate treatment [41,42,43,44]. On the other hand, K. Boengler et al. did not observe p66Shc translocation to mitochondria in ischemia/reperfusion injury, although ROS production was triggered [45]. In another study, though oxidative stress was induced by dihydrotestosterone (DHT) treatment in human prostate cancer cells, the translocation of p66Shc to mitochondria was not detected [46].

The abovementioned scientific literature shows that p66Shc protein localization may change depending on the conditions. In our study, we isolated ER-mitochondria contact sites (MAM fraction) to investigate the localization of p66Shc under basal and oxidative stress conditions. Using differential centrifugation and controlled proteolytic degradation, we found that p66Shc localizes predominantly to the MAM fraction in both animal (wild-type mouse liver) and cellular models (HepG2, HeLa, and 3T3/NIH cells). Our findings suggest that p66Shc is involved in mitochondrial function, with its localization to MAM particularly evident in healthy tissue and during oxidative stress. In addition, we comprehensively illustrated the distribution and the share of each ShcA isoform in fractions of mice livers and fractions isolated from HepG2 cells.

## 2. Results

### 2.1. Intracellular Localization of p66Shc in Mice Liver Tissue and HepG2 Cell Culture

**Figure 1:**
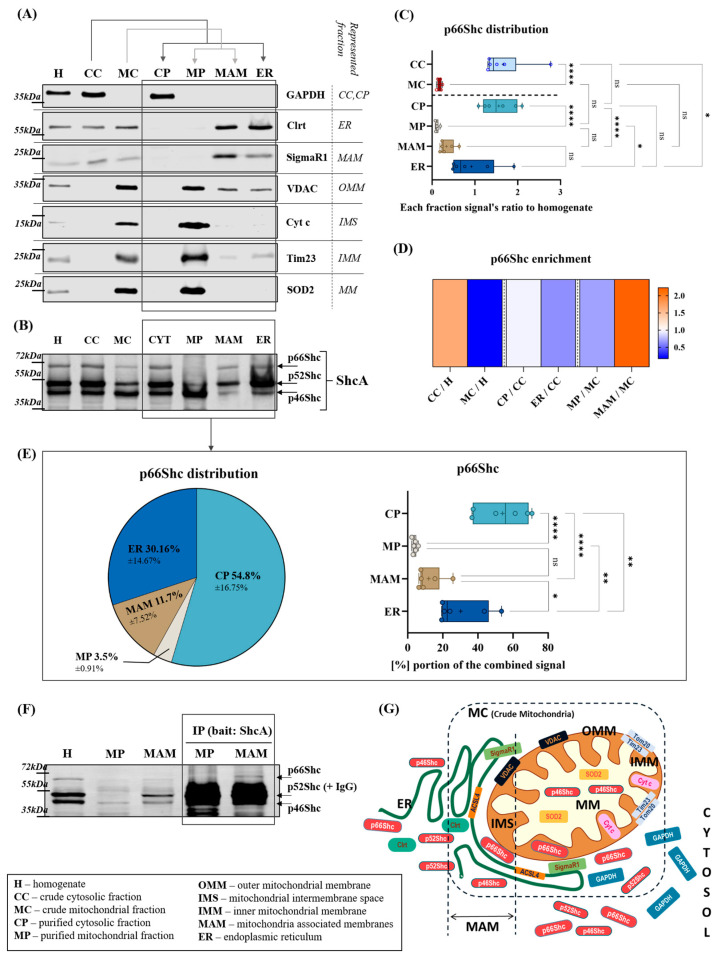
p66Shc distribution in cellular fractions isolated from mouse liver. (**A**) The levels of marker proteins in the fractions: for mitochondria (including crude mitochondria (MC) and pure mitochondria (MP))—mitochondrial superoxide dismutase (SOD2), voltage-dependent anion channel (VDAC), mitochondrial import inner membrane translocase (Tim23), and cytochrome c (Cyt c), for ER—calreticulin (Clrt), for mitochondria-associated membranes (MAM) - sigma non-opioid intracellular receptor 1 (Sigma R1), and for cytosol (including crude cytosol (CC) and pure cytosol (CP))—glyceraldehyde-3-phosphate dehydrogenase (GAPDH); (**B**) the level of ShcA proteins: p66Shc, p52Shc, and p46Shc in fractions isolated from mice livers shown in the representative Western blot picture; (**C**) quantification of p66Shc in each fraction compared to the p66Shc signal in total homogenate (H); box plots show the medians (lines), means are indicated with (+); *n* = 6; statistical significance evaluated with ordinary one-way ANOVA with Tukey’s method based multiple comparisons (**** *p* < 0.0001, * *p* < 0.05, ns—no significance); (**D**) heat map showing an enrichment of p66Shc in each fraction in reference to the fraction of origin: homogenate for crude cytosol and crude mitochondria, crude mitochondria for MAM, and purified mitochondria and crude cytosol for purified cytosol and ER; (**E**) graphs show the percentage contribution of p66Shc in each fraction in the total (100%) of p66Shc content calculated as a sum of p66Shc signals from MP, MAM, CP, and ER fractions (pie chart shows mean percentage representation of p66Shc in each fraction and box plot shows the medians (lines) and means indicated with (+) with SD); *n* = 6; Statistical significance evaluated with ordinary one-way ANOVA with Tukey’s method based multiple comparisons (**** *p* < 0.0001, ** *p* < 0.005, * *p* < 0.05, ns—no significance); (**F**) Western blot showing the levels of p66Shc in 25 µg of the total homogenate (H), pure mitochondria (MP), and MAM fractions samples as references and after immunoprecipitation (IP) with anti—ShcA antibodies in MP and MAM fractions from mice livers (IgG, heavy chain of the IP antibodies is visible together with p52Shc at approximately 52 kDa), *n* = 2; and (**G**) diagram illustrating the concept of the distribution of the investigated proteins across the intracellular fractions mentioned in the study, described in detail in the text.

We applied a multi-step approach to accurately examine p66Shc shares and localization in subcellular compartments. In the first step, we performed experiments using livers of the control C57/Bl6 mice and a procedure based on differential centrifugations allowing for a high level of purification of individual cellular fractions, as notably common procedures of mitochondria isolation end only with the “crude” mitochondrial (MC) fraction [47]. A schematic overview of the procedure is presented in Figure S1. In our experimental approach, to obtain a better insight into precise p66Shc distribution among subcellular compartments, we fractionated MC into highly purified mitochondria (pure mitochondria, MP) and MAM as indicated by arrows over the Western blot in Figure 1A and presented in detail in Supplementary Figure S1. 

To reliably assign the location of the ShcA isoforms, and especially the p66Shc protein, we had to verify the purity of the isolated fractions. For this reason, we first examined the enrichment of each fraction marker protein by Western blot. Marker proteins are the “housekeeping” proteins that typically localize in a specific intracellular compartment, and their localization is largely unaffected by experimental settings. In the present study, we used antibodies against the following marker proteins: glyceraldehyde-3-phosphate dehydrogenase (GAPDH) for cytosolic fraction (including CC and CP), calreticulin (Clrt) for ER fraction, sigma non-opioid intracellular receptor 1 (SigmaR1) for MAM fraction, cytochrome c (Cyt c), and superoxide dismutase 2 (SOD2) for mitochondria (including MC and MP). Furthermore, we evaluated the OMM and IMM markers, voltage-dependent anion channel (VDAC), and mitochondrial import inner membrane translocase (Tim23), respectively, when verifying the fraction purity. As shown in Figure 1A, GAPDH was present in the total homogenate sample (H) and enriched in crude cytosol (CC) and purified cytosol (CP), but not in any of the membranous compartments (ER, MAM, or mitochondria). Clrt, the protein typically localized in the ER lumen, was present in homogenate and both crude fractions, CC and MC, with significant enrichment in MAM and ER. MAM contains the portion of the ER fraction that interacts with mitochondria, while the majority of the ER fraction is contained in CC and separated from the cytosol by ultracentrifugation (see Supplementary Figure S1). SigmaR1 was present in H, CC, MC, and ER and enriched in MAM. Both mitochondrial markers, Cyt c, localized in mitochondrial intermembrane space (IMS), and SOD2 localized in the mitochondrial matrix (MM) were detected in the input samples, H and MC, with significant enrichment in pure mitochondria (MP). OMM and IMM markers are enriched in mitochondria, however, the detection of VDAC in MAM might be related to its involvement in the formation of a calcium-transferring protein complex in mitochondria–ER contact sites (between ER and mitochondria, with Grp75 as a tethering protein and inositol IP3R3 on the ER site). MAM is the fraction where we detect mitochondrial proteins (mostly assigned to the OMM) and ER proteins. The lack of ER and cytosolic markers in MP confirmed the mitochondrial fraction’s purity. The absence of mitochondrial markers in CP consistently demonstrated that both fractions were well separated and that the integrity of the mitochondria was maintained throughout the isolation process.

After verification of the quality of fractionation, we assessed the localization of p66Shc in the subcellular fractions. As shown in Figure 1B–D, among crude fractions, p66Shc was enriched in CC compared to its level in the input sample—H. The p66Shc pool in MC was smaller in comparison to CC. Among well-purified fractions, significant enrichment of p66Shc was visible in cytosol. As the detailed quantification of p66Shc presented in Figure 1E shows, if the four fractions resulting from subcellular fractionation are treated as 100%, most of p66Shc (over 54%) is localized to cytosol, almost 1/3 of the p66Shc content belongs to ER, approximately 11.85% is localized in MAM, and less than 4% in MP, and the p66Shc signal in MP was hardly detectable by Western blot. Knowing that the Western blot technique can be limited by the amount of the protein loaded on the gel, we tested rising amounts: 30 µg, 60 µg, and 90 µg of MP and MAM protein loaded on the SDS-PAGE. As shown in Figure S2, the level of p66Shc in MP, with the highest amount of protein loaded, did not exceed 10% of the level observed in 30 µg of the reference sample, (H) and its Western blot signal was still very low. Therefore, we tested whether immunoprecipitation with the use of specific anti-ShcA antibodies could increase the sensitivity of p66Shc detection in MP. As presented in Figure 1F, after pulling down, p66Shc was still almost undetectable in MP, whereas it was visible in MAM with detection comparable to the H sample. 

We performed a similar analysis for the shorter ShcA isoforms, p46Shc and p52Shc, which confirmed previous observations of mitochondrial p46Shc localization [3,4]; as presented in Figure S6A,C, the mitochondrial localization of p46Shc is dominant, however, a significant pool of p46Shc is also present in the cytosol. Less than 10% of p46Shc localizes in ER and MAM collectively (6.12% in ER and 3.04% in MAM). In contrast to p46Shc, as presented in Figure S6B,D, p52Shc localized mostly in the cytosol (over 57%), and over 31% was found in ER. MAM fraction contains less than 10% of p52Shc, and the least p52Shc contribution is mitochondrial (1.72%). 

In Figure 1G, we present a schematic illustration of the distribution of ShcA proteins in the compartments that this manuscript focuses on, i.e., mitochondria, MAM, ER, and cytosol. On the left, all abbreviations used in the figures, diagrams, and graphs to describe cellular compartments are defined. Additionally, the locations of proteins used as markers of given fractions are marked symbolically (Clrt, ACSL4 or SigmaR1, Tim23, VDAC, GAPDH, and SOD2). The dashed lines indicate areas isolated as crude mitochondria (MC) and MAM fractions. The presence of the aforementioned markers in the designated locations may be explained by the fact that these areas contain portions of both the mitochondrial and ER membranes. According to our concept, the p66Shc protein occurs in MAM, which is part of the MC fraction, a fraction usually isolated as functional mitochondria, which can explain why the p66Shc protein is often found in mitochondria (without excluding the possibility that a portion of p66Shc exists in or relocate to IMS). In addition, each of the ShcA isoforms presents a different enrichment pattern in isolated cell fractions. Accurate fractionation allows for the description of the content of individual ShcA proteins in cellular compartments in contrast to visualization by microscopy after labeling the peptide with antibodies, which recognize domains common for all isoforms. Thanks to this, we can also observe that the individual isoforms’ pattern is significantly repeatable in samples of control mice liver homogenates. We assessed that p66Shc contributes in approx. 3.6% to the total ShcA pool, while p46Shc contributes 25.7% and p52Shc is the dominant ShcA isoform which contributes in 70.6% to the total ShcA as shown in Figure S6E.

**Figure 2:**
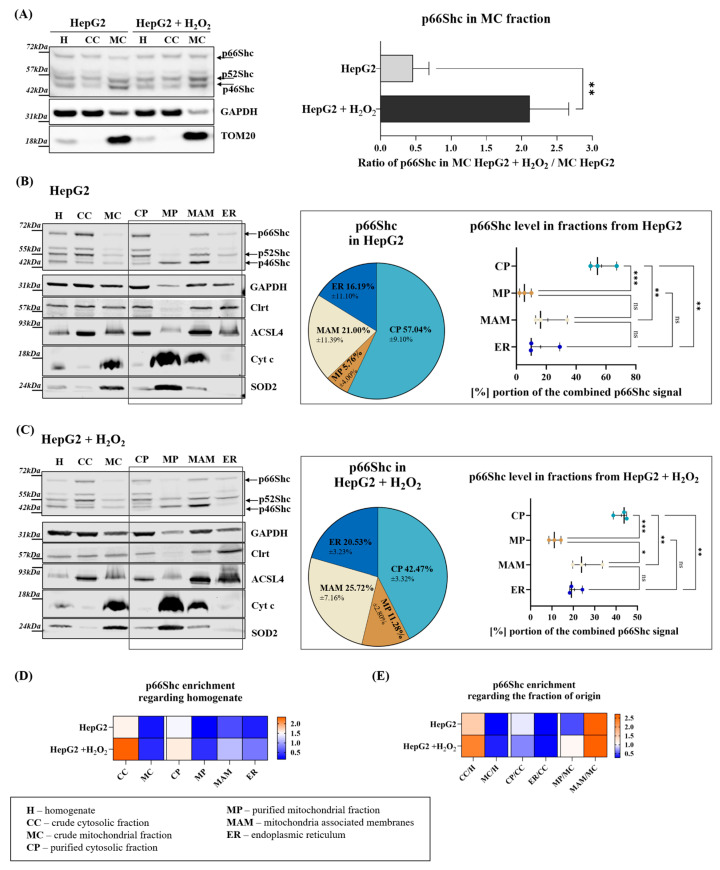
p66Shc distribution in cellular fractions isolated from HepG2 cell cultures. (**A**) A comparison of the p66Shc protein levels in crude fractions: homogenate (H), post-mitochondrial supernatant—crude cytosolic fraction (CC), and crude mitochondrial fraction (MC) in HepG2 cells untreated and treated with the H_2_O_2_ followed by the quantification of p66Shc ratios between MC from H_2_O_2_ treated HepG2 to untreated cells after normalization to the level in the total homogenate (input) samples; *t*-test ** *p* < 0.005; (**B**,**C**) the levels of ShcA proteins: p66Shc, p52Shc, and p46Shc in fractions isolated from untreated HepG2 cells (**B**) and in fractions isolated from HepG2 cells treated with 1 mM H_2_O_2_ for 24 h (**C**) followed by p66Shc fraction shares quantification showed as the percentage contribution of p66Shc in each fraction in the total (100%) of p66Shc content calculated as a sum of p66Shc signals from MP, MAM, CP, and ER fractions; pie charts show mean percentage representation of p66Shc in each fraction and respective box plots show the mean with (SD); plots show the median with SD, means are indicated with (+); statistical significance evaluated with ordinary one-way ANOVA with Tukey’s method based multiple comparisons (*** *p* < 0.0005, ** *p* < 0.005, and * *p* < 0.05, ns—no significance); levels of marker proteins: for mitochondria—mitochondrial superoxide dismutase (SOD2) and cytochrome c (Cyt c), ER—calreticulin (Clrt), MAM: long-chain-fatty-acid-CoA ligase 4 (ACSL4), cytosol (CC and CP)—GAPDH; (**D**) heat map presenting quantification of a p66Shc ratio in each fraction to p66Shc in homogenate; and (**E**) heat map presenting quantification of a p66Shc ratio in each fraction to p66Shc in the respective fraction of origin: homogenate for MC and CC, CC for ER and cytosol, and MC for MAM and MP.

Since the p66Shc level in mitochondria increases after oxidative stress induction [10,11,41], we decided to describe the precise intracellular localization of p66Shc in stress conditions. H_2_O_2_ is a well-established, commonly used, and easy-to-handle reagent with pro-oxidant properties; moreover, it is known to promote p66Shc translocation to mitochondria [11]. For this purpose, we used cell cultures instead of animal tissue, which has limitations in terms of oxidative stress induction. Separation of subcellular fractions from cell cultures is much more demanding than from tissue due to low efficiency and requires optimization concerning the original protocol. Therefore, we used the HepG2 cell line, which grows relatively efficiently and allowed us to obtain a minimal sufficient amount of material for isolation and fractions for performing assays, such as controlled digestion followed by Western blot. Based on the available data on the range of H_2_O_2_ concentrations that promote oxidative stress and activate the p66Shc-related pathway, we decided to treat the cells with 1 mM H_2_O_2_ in culture media for 24 h. After this treatment, we observed an increase in the MitoSOX™ probe oxidation by approx. 30%, decreasing survival by approx. 20% and increasing levels of cleaved poly [ADP-ribose] polymerase (PARP) and serine 36 phosphorylated p66Shc as presented in Figure S3. Though higher concentrations efficiently led to a substantial cell death rate increase in the culture, such conditions would create difficulties in obtaining enough material and isolating high-quality fractions from cells seriously affected by cell death. Selected conditions promoted the double increase in the p66Shc level in MC fraction in HepG2 cells treated with 1 mM H_2_O_2_ compared to MC isolated from untreated cells as presented in Figure 2A.

Similarly, as in the case of experiments on mice livers, we isolated purified subcellular fractions from HepG2, in which, in the first step, we verified organelles markers using Western blot. As shown in Figure 2B,C, both in fractions isolated from untreated and H_2_O_2_-treated HepG2, GAPDH was enriched in CC and CP, Clrt in ER, long-chain-fatty-acid-CoA ligase 4 (ACSL4) typically localized in ER interacting with mitochondria was enriched in MAM, Cyt c, and SOD2 were enriched in mitochondria (in MC and MP). However, in this case, mitochondrial markers were also visible in the cytosolic fraction, as well as low amounts of GAPDH were present in all fractions, indicating particular difficulties with avoiding cross-contamination while isolating subcellular fractions from cell cultures. Nevertheless, as pie charts and respective box plots in Figure 2B,C show, p66Shc distribution was similar to the pattern observed in the case of mice liver fractions. 

In both conditions (untreated and H_2_O_2_ treated cells), p66Shc was enriched in CC (Figure 2D,E), and subsequently, its highest amount among purified fractions was observed in CP. There was less p66Shc in MC than in CC, however, in contrast to mice livers, it was well visible in MAM and detectible in mitochondria by Western blot without a need for precipitation. In contrast to the liver samples, in HepG2 fractions, MAM p66Shc content was higher than in the ER. In fractions from untreated cells, the p66Shc share in MP was approx. 5.8% of the total p66Shc in all four isolated fractions (treated as 100%); however, after H_2_O_2_, it increased almost twice. As presented by the pie charts in Figure 2B,C, after H_2_O_2_, cytosolic p66Shc contribution decreased by approximately 15% in favor of the ER, MAM, and MP shares. MAM and ER p66Shc shares were increased in oxidative stress conditions by less than 5%. Analysis of the p66Shc enrichment, when quantified as a ratio of the level in each fraction to the level detected in homogenate (total fractionation input), shown in Figure 2D, and as a ratio of p66Shc in each purified fraction to the respective input fraction, Figure 2E (CC and MC to homogenate, MP, and MAM to MC, and ER and CP to CC) shows that both under control and oxidative stress conditions, the cytosol is the primary p66Shc localization, and next it is ER and MAM. Mitochondrial p66Shc signal is depleted when referred, both to homogenate and to the crude mitochondria. 

As in the case of mice liver fractions, we also assessed the distribution of the shorter ShcA isoforms, p46Shc and p52Shc, in HepG2 cells. When the levels of either p46Shc or p52Shc in each analyzed fraction (CC, MC, CP, MP, MAM, and ER) were expressed as a ratio to their levels in homogenate, there were no statistically significant differences. Nevertheless, the mitochondrial fraction was a predominant localization site for p46Shc both in control and H_2_O_2_-treated HepG2 cells as shown in Figure S7A,B. In contrast to p46Shc, in both conditions, p52Shc was localized mostly in the cytosol. There was more p52Shc in MAM after H_2_O_2_ treatment than in control conditions; however, the differences were not significant. When we focused on the distribution of p46Shc and p52Shc among purified cytosol (CP), purified mitochondria (MP), MAM, and ER fractions, we confirmed mostly mitochondrial p46Shc localization, and mostly cytosolic p52Shc localization (Figure S7C,D). In control conditions, the differences in the distribution of p46Shc and p52Shc between pure cytosol (CP) and pure mitochondria (MP) were statistically significant. The distribution of p46Shc after the H_2_O_2_ treatment was similar to control conditions, and the differences between its levels in MP and CP, and MP and ER were statistically significant in contrast to p52Shc. The graph presented in Supplementary Figure S7E shows that the share of each isoform in the total ShcA signal in homogenates of HepG2 cells does not change significantly upon oxidative stress. Though the differences between the shares of individual isoforms are not significant, we noticed that in control HepG2 cells, the percentage contribution of p46Shc and p66Shc are similar: 25.175% and 24.054%, respectively. p52Shc, similarly to liver homogenates, is the dominant ShcA isoform both in control and H_2_O_2_-treated HepG2 cells.

### 2.2. Trypsin Digestion of p66Shc Localized in “Crude” Mitochondria

Subcellular fractionation data from HepG2 cell cultures and mouse livers indicate that p66Shc is mostly found in the cytosol and ER. p66Shc content in MC when divided into MAM and MP seems to be mostly attributed to its MAM localization. MC fraction contains multiple membranous organelles’ fragments which interact with mitochondria. Therefore, we decided to verify with another method, the exact p66Shc origin in MC. In this step, a controlled digestion with increasing concentrations of trypsin was used. A similar approach involving proteolysis was previously used to verify the mitochondrial localization of, e.g., ERK [48]. 

A total of 100 µg of crude mitochondrial fraction (MC) from HepG2 cells, untreated and treated with 1mM H_2_O_2_, were subjected to rising trypsin concentrations to establish a point at which p66Shc disappears. Gradual controlled trypsinization was intended to verify whether the p66Shc protein would be digested as MAM markers or together with mitochondrial markers. MC isolated from unstimulated HepG2 cells were treated with 0.5 μg, 1 μg, 2 μg, 5 μg, 10 μg, and 25 μg per 1 mL of reaction mixture for 15 min. With the selected trypsin concentration, we achieved a gradual, close-to-linear decrease in protein content in the samples as verified by PVDF membrane Ponceau staining after SDS-PAGE separation and transfer of trypsinized MC samples as shown in Figure S4A. 

**Figure 3:**
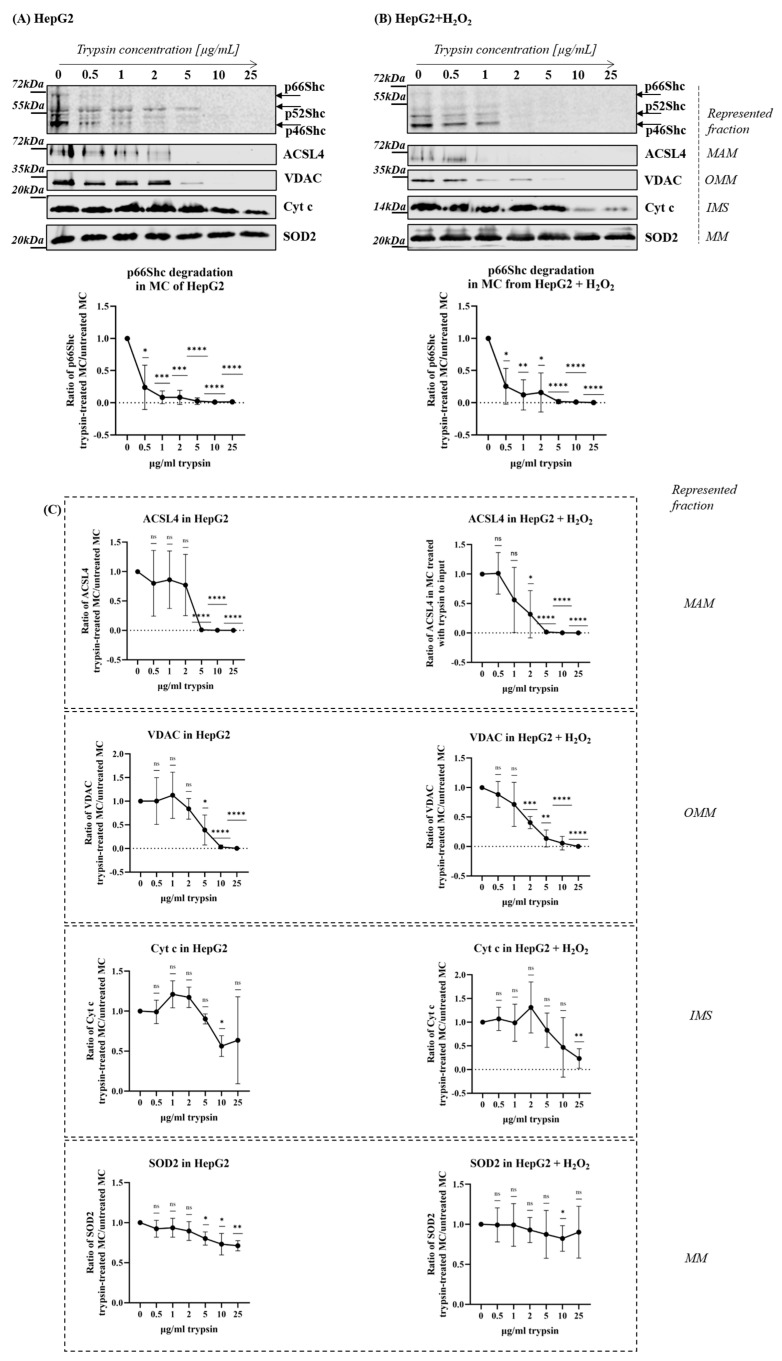
p66Shc digestion with trypsin in crude mitochondrial fraction isolated from the HepG2 cell line. p66Shc protein and fractions markers: SigmaR1 for MAM, VDAC for outer mitochondrial membrane (OMM), Cyt c for intermembrane space (IMS), and SOD2 for mitochondrial matrix (MM) detected by Western blot in the residual samples after trypsin digestion of 100 µg of MC isolated from (**A**) control untreated HepG2, and (**B**) HepG2 cells treated with 1 mM H_2_O_2_ for 24 h. Quantification presented below the representative Western blots shows the mean ratio of p66Shc (**A**,**B**) and fractions markers (**C**) in trypsinized samples to the level of p66Shc (**A**,**B**) or each fraction marker (**C**) in the input untreated MC sample (value is assigned as 1) with SD. Statistical significance was calculated with one sample *t*-test (where value = 1 refers to the input—undigested MC sample), *p*-value (**** *p* < 0.0001, *** *p* < 0.0005, and ** *p* < 0.005, * *p* < 0.05, ns—no significance).

Presented in Figure 3A, quantification of the Western blot analysis of ShcA proteins in MC after the proteolysis revealed that the p66Shc signal decreased significantly by approximately 50% of the initial level already after 0.5 μg/mL trypsin, and after 2 μg/mL of trypsin, it became undetectable, while ACSL4, the marker of MAM, was still present (Figure 3B). VDAC, the marker of OMM, started to be digested at the 5 μg/mL of trypsin, but Cyt c resisted up to 10 μg/mL of trypsin used. SOD2, localized mostly in the mitochondrial matrix, and used as a mitochondrial marker, was decreased by approximately 25% with 10 μg/mL trypsin. This result shows that most of the p66Shc protein pool was digested before the mitochondria were disrupted, which suggests it is mostly present outside of mitochondria, and therefore, in the MAM fraction. At this point, it should be stressed that we are aware of the soluble nature of the p66Shc in contrast to VDAC or ACSL4, which are membrane proteins, which may affect their availability for trypsinization. However, as we observed Cyt c, a soluble IMS protein required a higher trypsin concentration and resisted much longer than the p66Shc and OMM markers (Figure 3B).

As shown in Figure 2C, after treatment of HepG2 cells with H_2_O_2_, we observed an increase in the p66Shc pool in MAM and mitochondrial fractions. Knowing that those two fractions are derived from crude mitochondria (MC) fraction, in the next step, we verified whether controlled trypsinization will suggest that, after p66Shc relocalization between subcellular compartments induced by oxidative stress, the p66Shc contribution in MC could also be of MAM origin, similar to control conditions in the panel A of Figure 3. As presented in Figure 3B, as in the case of untreated HepG2, the p66Shc level decreased significantly already after digestion with 0.5 μg/mL trypsin, and with 5 μg/mL trypsin, it was not detectable. MAM and OMM marker (ACSL4 and VDAC) decreased levels were observed after 2 μg/mL. Cyt c, the IMS protein, instead was significantly digested only with the highest trypsin concentration (25 μg/mL). This suggests that, even though we stress the cells to induce p66Shc translocation to mitochondria, most of the p66Shc can be still detected in MAM. 

To increase the sensitivity of p66Shc detection and to verify whether, after 5 μg/mL trypsin, any p66Shc can be further detected, we performed digestion of 100 µg of MC with 1 µg/mL and 5 µg/mL trypsin for 15 min. After stopping the reaction, we performed IP with anti-SHCA antibodies to pull down the residual pool of p66Shc. Western blot analysis with anti-ShcA antibodies, presented in Figure S4B, shows that the signal from p66Shc both in untreated and H_2_O_2_-stressed HepG2 after 1 μg/mL trypsin was very low, and after 5 μg/mL, was hardly detectible with this technique. In line with previous results, a relatively low p66Shc signal, which was observed after 1 and 5 µg of trypsin, suggested that most of the p66Shc pool disappeared before the IMS marker Cyt c was degraded. This supports our findings that the MAM fraction isolated from HepG2 contains a significant amount of p66Shc protein in comparison to mitochondria. 

We also performed controlled digestion on HeLa and 3T3-NIH fibroblasts, as well as on fractions derived from mouse livers, to optimize the experimental design before HepG2 cells were tested. These analyses’ results, which are shown in Figure S5, align with the findings of the analysis conducted on the HepG2 cell line (Figure 3). MC isolated from mouse liver was treated with the rising concentrations of trypsin: 10 μg/mL, 25 μg/mL, 50 μg/mL, 75 μg/mL, and 100 μg/mL (much higher than in the case of cell cultures) for one hour (Figure S5A), or with 50 μg/mL in a time-dependent manner for 10, 20, 30, 40, and 60 min (Figure S5B), followed by the detection of ShcA proteins and markers SigmaR1 for MAM, VDAC for the outer mitochondrial membrane (OMM), Cyt c for the mitochondrial intermembrane space (IMS), and SOD2 for mitochondrial matrix (MM). In these conditions, we observed continuous, but not complete, degradation of p66Shc similar to the MAM marker. 3T3-fibroblasts and HeLa cells were stressed with H_2_O_2_ to promote mitochondrial p66Shc localization, after that, we isolated MC fraction, which we used for trypsinization (concentration range 10–200 μg/mL) followed by p66Shc analysis with Western blot. In both HeLa (Figure S5C) and 3T3-NIH (Figure S5D), p66Shc was digested with the lowest trypsin concentration, similarly to the MAM marker, while the OMM marker was slightly affected and IMS protein Cyt c level was unchanged. 

These findings imply that the cellular distribution of the p66Shc protein may depend on both the model studied and the experimental conditions. The biochemical approach to verifying the protein localization in the cell should be carefully tailored to the type of material, as our results suggest differences in the properties and compatibility of the proteins for the controlled digestion assay. Nevertheless, similarities in the digestion patterns of the p66Shc protein in different models suggest its primary extramitochondrial MAM localization.

## 3. Discussion

p66Shc is a protein involved in various cellular processes determining cell fate. Many reports show an increase in mitochondrial content of p66Shc upon oxidative stress, followed by an apoptosis activation due to the accumulation of oxidative damage and the antioxidant response collapse. Most studies refer to p66Shc as a redox protein localized in the cytosol, ER, or mitochondria, involved in pathologies in which oxidative stress is a key factor [49,50,51,52]. However, there is no consensus on the p66Shc localization because of differences in approaches to subcellular fractions isolation and investigation. Interestingly, there are visible differences in the distribution of the three ShcA isoforms among the subcellular compartments. Compartmentalization of the isoforms may determine their participation in signaling pathways, primary functions, and interactions. Precise investigation of each of the ShcA isoforms’ features is associated with several complications due to their structure and how the gene transcript and then polypeptide is generated. A single DNA locus is the source of all three Shc isoforms. Alternative splicing produces two mRNA (p66Shc and p52/46Shc), but all three Shc isoforms have start codons on the p66Shc mRNA. Only p52Shc and p46Shc are produced by the p52/46Shc-mRNA, which lacks a start codon for p66Shc. Because of this, knockdowns of p66Shc or all three Shc isoforms have been accomplished [4,53]. What is more intriguing and may affect the understanding of each ShcA isoform mode of functioning is that, already in 2004, Ventura and colleagues reported that p46Shc localizes to the mitochondrial matrix because it contains an internally localized mitochondrial targeting sequence that is inactive in p52Shc and p66Shc [3] rising questions of their localization dynamics and functions. In our studies, we confirmed the previous findings on predominant localizations of each of the ShcA isoforms; however, we also precisely described that the shares of individual isoforms, both among fractions and in the total ShcA pool in different conditions, control mice livers and hepatocellular carcinoma cells untreated and after H_2_O_2_ stimulation. In light of previous literature data on ShcA adaptors’ roles in cancer, our observations may suggest that further studies on changes in ShcA isoform distribution patterns may help to understand oncogenesis-related signaling.

Thus, one of our aims was to confirm the previous observation of p66Shc presence in MAM. MAM is a structural and functional hub where proteins even allocated to various subcellular compartments may interact to evoke certain metabolic effects or activate processes such as apoptosis, e.g., by promoting calcium release from the ER and uptake by mitochondria [31]. By modulating ROS levels and mitochondrial function, p66Shc can influence cell survival decisions. In mitochondria, p66Shc can shuttle electrons from cytochrome c to molecular oxygen, generating ROS and contributing to oxidative stress [24]. However, p66Shc in MAM could interact with mitochondrial proteins and regulate mitochondrial functions and oxidative stress response. Mitochondria–ER contact sites, often called MAM, are specialized regions playing crucial roles in various cellular processes, including lipid metabolism, calcium signaling, and mitochondrial dynamics. The dynamic interplay between mitochondria and the ER at these contact sites is essential for maintaining cellular homeostasis and responding to extra- or intracellular stressors [54]. We previously reported a shift in p66Shc from plasma membrane-associated membranes, where it participates in mitotic signal transduction, to MAM, where it can be dedicated to oxidative stress response regulation in the livers of aged mice. However, the distribution of p66Shc between the fractions of a control animal was not quantified at that time [20]. As mentioned previously, not in all reports, p66Shc has been observed to be translocated to the mitochondria [45,46]. These differences may be related to the experimental model, for example, the type of cells or tissue, but also to the technique used to isolate the mitochondrial fraction. We noticed that when investigating the mitochondrial translocation of the p66Shc protein upon stress, most authors [24,41,55,56,57] prepare mitochondrial fraction in a way that yields “crude mitochondria”, a fraction containing not only mitochondria but also membranes of the other organelles. Mitochondrial fraction prepared in this way is particularly useful in functional studies [47]; however, to study precise localization, well-purified factions are crucial. The basic protocol for mitochondria isolation, which consists of a combination of homogenate centrifugations (slow (up to 700× *g*, 5 min) followed by fast one (10,000× *g*, 10 min)), does not lead to the disruption of interactions between mitochondria and other membranes. For this reason, MC contains other organelles such as ER or even plasma membrane fragments [58,59]. To separate mitochondria from MAM, as indicated in Figure S1, ultracentrifugation (95,000× *g*, 30 min) in Percoll^®^ gradient is required as described in detail in the protocol by Wieckowski et al., 2009 [59]. Using this approach, we obtain a well-purified fraction of mitochondria and separate mitochondria-associated membranes fraction. Moreover, we can also roughly separate cytosol and microsomes (mostly ER membranes) by performing ultracentrifugation of the “crude” cytosolic fraction. Additionally, in many studies described in the literature, commercially available organelle isolation kits are used. In these kits, buffers or reagents are not adjusted to the material used. Some of those kits are based on the use of detergent (e.g., digitonin) to permeabilize plasma membrane; therefore, when non-adjusted, they may result in uncontrolled protein leak between the permeabilized compartments. In another common technique to study localization, which is based on confocal microscopy, p66Shc, and mitochondrial signals overlay, which leads to a conclusion that p66Shc is localized in mitochondria. However, verification of a p66Shc presence in mitochondria under physiological conditions without genetic manipulation is limited since antibodies available at the market recognize all three ShcA isoforms (p66Shc, p52Shc, and p46Shc). For these reasons, we have chosen a well-standardized protocol for fractions isolation to compare the range of p66Shc enrichment between the cytosolic, ER, mitochondrial, and MAM fractions in a control C57/Bl6 mouse liver and cellular model HepG2 cells. Our data contribute to the knowledge of p66Shc localization in these two popular in vivo and in vitro experimental models. We are aware that it would be useful to verify these observations in other models often used in studies on p66Shc-related pathologies. However, for this purpose, an optimization of the isolation procedures is required to obtain a reliable amount of MAM material, which is particularly tricky in the case of cell cultures. In the present study, we show that, despite the difference in HepG2 cells and mouse liver tissue, we can efficiently isolate MAM and the distribution of p66Shc among fractions is comparable in the HepG2 cell line and mouse liver fractions. Moreover, consistent with the literature data, we observed redistribution and an increase in mitochondrial and MAM content of p66Shc among the fractions in HepG2 cells after oxidative stress induction. Based on the results, we cannot conclude whether we detect more p66Shc in MAM because it is “caught” on its way to mitochondria. There is no perfect method to separate the subcellular compartments, which may raise the scientific question of whether the p66Shc observed in MAM similar to p66Shc observed in mitochondria contributes to the redox balance disruption. Nevertheless, since in control and oxidative stress conditions a significant pool of p66Shc protein is present in MAMs, it may be exactly a niche for p66Shc. Mitochondria–ER contact sites create a permissive environment for p66Shc to interact with mitochondrial proteins, enabling modulation of mitochondrial functions. In our study, we combined the available traditional methods based on differential centrifugation and increased their sensitivity by pulling down the traces of the protein in mitochondrial and MAM fractions. Immunoprecipitation performed to increase the sensitivity of p66Shc detection proved that only its trace is detectible in mitochondria, while we detect most of p66Shc in MAM. We focused on the four main subcellular compartments, where p66Shc was reported to be localized: cytosol, ER, MAM, and mitochondria. Additionally, though we were not able to answer whether exactly the MAM pool of p66Shc affects the mitochondrial function, after analysis of the results presented in this paper, we can conclude that the amount of protein in mitochondria is relatively low and that MAM contains a greater amount of p66Shc. Moreover, the literature mentions some protein candidates localized in the OMM that could possibly participate in the p66Shc-mediated ROS production, e.g., NADH-cytochrome b5 reductase 3 (CYB5R3) [24].

Overall, the localization of p66Shc in MAM underscores its role in regulating mitochondrial function, oxidative stress, and apoptosis. Understanding the dynamics of p66Shc at these specialized membrane contact sites provides insights into its physiological and pathological roles in cellular homeostasis and disease. To conclude, our results appear to confirm that the cellular distribution of the p66Shc protein is related to the studied model and conditions; nevertheless, we provided a piece of evidence for p66Shc localization in MAM and described ShcA isoforms distribution among cytosolic, endoplasmic reticulum, mitochondria-associated membranes, and mitochondrial fractions. 

## 4. Materials and Methods

### 4.1. Subcellular Fractionation of Mouse Liver and HepG2 Cells

1. C57/Bl6 mice (males) were anesthetized with isoflurane inhalation and sacrificed by cervical dislocation. Livers were immediately excised and washed in a cold phosphate-buffered saline (PBS) to remove remaining blood and then homogenized in a pre-cooled homogenization buffer and homogenized in a motor-driven Potter-Elvehjem homogenizer with PTFE pestle as described in Wieckowski et al. [59].
2. HepG2 cells, 3T3/NIH fibroblasts, and HeLa cells (ATCC, Manassas, VA, USA) were grown in Dulbecco’s modified Eagle’s medium (DMEM) (Lonza Bioscience, Basel, Switzerland), supplemented with 10% fetal bovine serum (FBS) (Gibco, Thermo Fisher Scientific, Waltham, MA, USA), L-glutamine (Sigma-Aldrich, St. Louis, MO, USA), and penicillin/streptomycin solution (Sigma-Aldrich, St. Louis, MO, USA) in 15 cm Ø plates. Selected plates were treated with 1 mM H_2_O_2_ (Sigma-Aldrich, St. Louis, MO, USA) in a serum-free medium for 24 h before fractionation. On the day of the experiment, cells were detached using a cell scraper, centrifuged at 200× *g* for 3 min, and washed twice with PBS. Afterward, the cell pellet was resuspended in the cold homogenization buffer (Wieckowski et al. [59]) and homogenized in a tightly fitting glass–glass Potter-Elvehjem homogenizer.

Homogenates were centrifuged twice at 600× *g* for 3 min at 4 °C to pull down the unbroken cells and nuclei. Supernatants were centrifuged at 10,000× *g* for 10 min to obtain “crude” mitochondrial (MC) fraction and “crude” cytosolic fraction (CC). MC and CC fractions were used for further fractionation according to the procedure based on differential centrifugation in Percoll^®^ (Sigma-Aldrich, St. Louis, MO, USA) gradient described by Wieckowski et al. [59]. 

Prepared as described above, the MC fraction was also used for proteolytic degradation experiments. 

### 4.2. MitoSOX™ Oxidation and Cell Mass Assessment

To verify the upregulation of reactive oxygen species production in H_2_O_2_-treated HepG2 culture, cells were seeded in the 24-well plate and grown for 24 h in control DMEM supplemented with 10% FBS or in the absence of FBS in media and with an addition of 0.5 mM, 1 mM, and 2 mM H_2_O_2_. After incubation, cells were washed with phosphate-buffered saline (PBS) twice followed by an addition of 5 µM MitoSOX™ (Invitrogen, Thermo Fisher Scientific, Waltham, MA, USA) fluorescent probe in PBS with 25 mM glucose for 10 min. Afterwards, cells were washed twice with PBS and a fresh solution of 25 mM glucose-containing PBS was added to the wells. A MitoSOX™ fluorescent signal was detected with 510 nm excitation and 595 nm emission wavelengths in the Tecan Infinite M200 (Tecan, Grödig, Austria) multiwell plate reader. Then, the PBS solution was discarded and the cells in the wells were fixed with ice-cold 5% acetic acid in methanol for 24 h. Fixed cells were then dried and stained with 0.4% sulforhodamine B (SRB) (Sigma-Aldrich, St. Louis, MO, USA) in 1% acetic acid solution for 1 h. After this time, staining solution was discarded and the dye excess was washed out with 1% acetic acid and dried. The SRB stain was washed out from cells by an addition of 10 mM Tris. An absorbance of a colored solution in the plate was measured at 540 nm wavelength using a Tecan Infinite M200 reader. Results of fluorescence and absorbance measurements were processed using Magellan 7.2 (Tecan, Grödig, Austria) software.

### 4.3. Trypsin Digestion of “Crude” Mitochondria-Associated p66Shc

A stock solution of 100 mg/mL trypsin (Sigma-Aldrich, St. Louis, MO, USA) in PBS was diluted in the reaction tubes to the desired final concentrations (as indicated in Section 2.2 and Figure 3, Figures S4 and S5 legends) with the homogenization buffer, and the reaction was started by the addition of 100 µg of the MC. The digestion was stopped by adding a protease inhibitor cocktail (Thermo Fisher Scientific, Waltham, MA, USA). Then, the samples were centrifuged at 16,000× *g* for 10 min to pellet the residual undigested fraction. Supernatants were discarded, and the pellets were resuspended in a cold RIPA buffer (Sigma-Aldrich, St. Louis, MO, USA). After 15 min of incubation on ice, samples were centrifuged again at 16,000× *g* for 15 min. The pellets were discarded and supernatants were transferred to the new tubes, followed by 4× Laemmli sample buffer addition. Samples were then denatured at 95 °C for 5 min and processed with the Western blot technique.

### 4.4. Immunoprecipitation of p66Shc

Samples were resuspended in the cold immunoprecipitation (IP)–lysis buffer containing 25 mM Tris-HCl, pH 7.5, 100 mM NaCl, 1 mM EDTA, 1% glycerol, and 0.1% Igepal^®^ (Sigma-Aldrich, St. Louis, MO, USA) and incubated on ice for 20 min. Next, lysates were centrifuged at 16,000× *g* for 15 min. Protein concentration in supernatants was determined using Bio-Rad Protein Assay Dye Reagent Concentrate (Bio-Rad, Hercules, CA, USA). p66Shc protein was precipitated by the incubation of 200 µg protein of total lysate (or residual fraction from trypsinization) with IP-dedicated anti-SHCA antibodies (Bioss, Woburn, MA, USA) on the agarose A/G protein-conjugated beads (Santa Cruz Biotechnology, Dallas, TX, USA) overnight at 4 °C with gentle mixing. Precipitates of p66Shc protein immobilized on agarose beads were washed 3 times in the cold IP buffer to remove nonspecific interactions. Proteins were eluted from beads in denaturing conditions by the addition of 1× Laemmli buffer and incubation at 95 °C for 5 min and analyzed by Western blot.

### 4.5. SDS-PAGE and Western Blot

Subcellular fractions’ samples were resuspended in the cold RIPA buffer (Sigma-Aldrich, St. Louis, MO, USA) supplemented with a protease and phosphatase inhibitors cocktail (Thermo Fisher Scientific, Waltham, MA, USA). After incubation on ice, samples were centrifuged at 16,000× *g* for 15 min. Protein concentration in the lysates was measured with Bio-Rad Protein Assay Dye Reagent Concentrate (Bio-Rad, Hercules, CA, USA). Samples intended for sodium dodecyl sulfate-polyacrylamide gel electrophoresis (SDS–PAGE) were denatured with 4× Laemmli loading buffer at 95 °C for 5 min. Samples were separated in 10% (for over 25 kDa targets) or 13% (for low molecular weight targets) hand-casted polyacrylamide gels (Bio-Rad, Hercules, CA, USA) and transferred onto polyvinylidene fluoride (PVDF) membranes (Bio-Rad, Hercules, CA, USA). Protein loading was verified with Ponceau staining. Membranes were blocked using 5% non-fat milk/blocking grade (Bio-Rad, Hercules, CA, USA) in Tris-buffered saline (TBS) supplemented with 0.01% Tween-20 (Sigma-Aldrich, St. Louis, MO, USA) for 1 h. The membranes were then incubated overnight at 4 °C with the primary antibodies followed by incubation with the proper anti-mouse or anti-rabbit secondary HRP-conjugated (Santa Cruz Biotechnology, Dallas, TX, USA) or fluorescent IRDye (Li-Cor, Biosciences, Lincoln, NE, USA) antibodies. The chemiluminescence was detected using an ECL kit (GE Healthcare Bio-Sciences, Piscataway, NJ, USA) in X-ray film processing equipment. The fluorescent signal was detected using an Odyssey infrared imaging system (Li-Cor, Biosciences, Lincoln, NE, USA) or ChemiDoc MP (Bio-Rad, Hercules, CA, USA) scanner. Bands’ intensities were evaluated using Image Studio Lite software version 5.2 (Li-Cor, Biosciences, Lincoln, NE, USA) or Image Lab 6.1.0 (Bio-Rad Laboratories Inc., Hercules, CA, USA).

### 4.6. Data Analysis

Qualitative and quantitative data from Western blot analyses were processed in the Image Studio Lite software version 5.2 (Li-Cor, Biosciences, Lincoln, NE, USA) or Image Lab (Bio-Rad, Hercules, CA, USA) and processed for calculation in Excel 2019 (Microsoft, Redmond, WA, USA). Spectrophotometric and fluorimetric data were processed in Magellan 7.2 (Tecan, Grödig, Austria) and analyzed in Excel 2019 (Microsoft, Redmond, WA, USA). Graphs present mean values from at least 3 biological replicates (if otherwise described under the figures) with standard deviations (SD). Statistical analysis was performed using GraphPad 10.3.1 (Prism, Boston, MA, USA).

## Data Availability

The original contributions presented in the study are included in the article/Supplementary Material, further inquiries can be directed to the corresponding author.

## Supplementary Materials

The supporting information can be downloaded at: https://www.mdpi.com/article/10.3390/ijms252312835/s1.
