# Supplementary material for: Distribution of the p66Shc Adaptor Protein Among Mitochondrial and Mitochondria—Associated Membranes Fractions in Normal and Oxidative Stress Conditions
Source: Int J Mol Sci. 2024 Nov 29;25(23):12835. doi: 10.3390/ijms252312835 (PMC11640770; doi:10.3390/ijms252312835)
Supplement: Supplementary file 1 [file ijms-25-12835-s001.zip › ijms-3310483-supplementary.pdf]

Supplementary Figures:

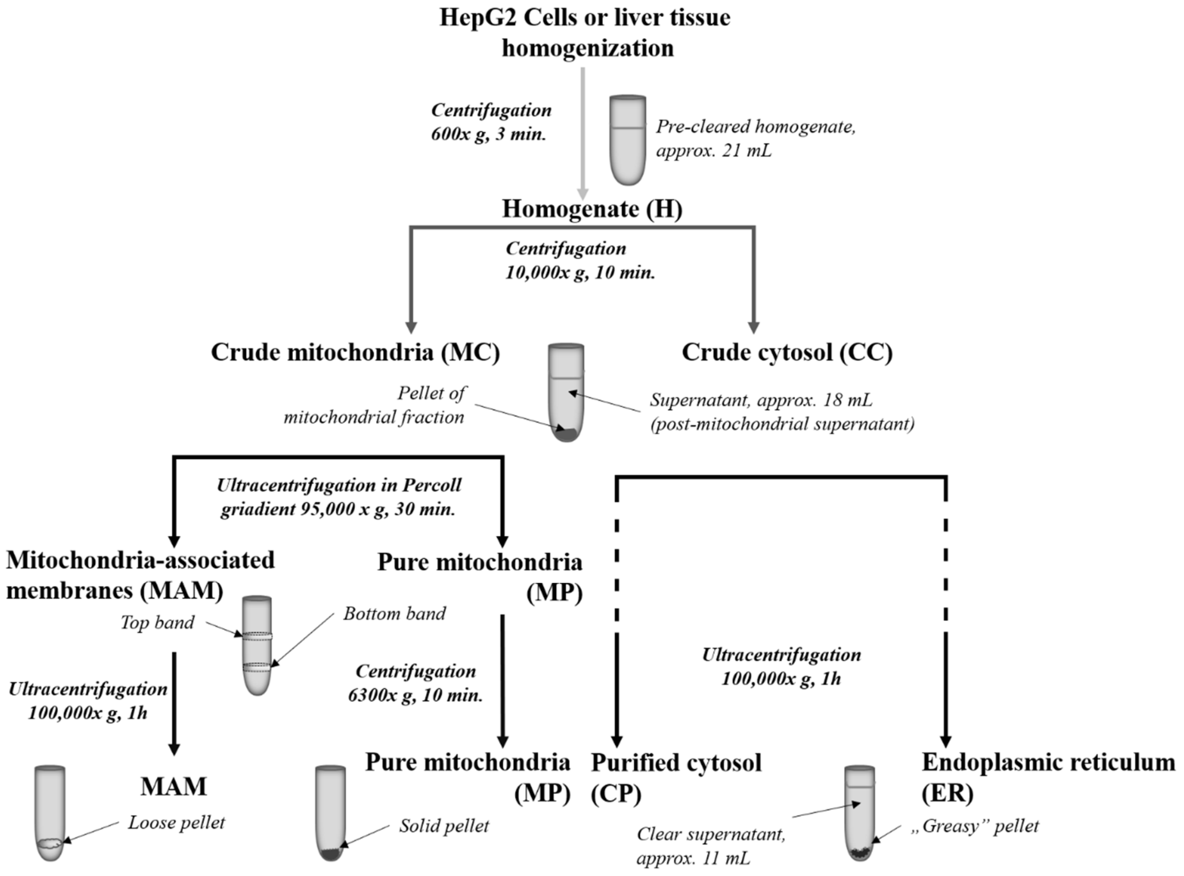

Figure S1: Diagram illustrating the main steps in the fractionation protocol applied in the study.

Homogenate (H) prepared from liver tissue or cell cultures (HepG2) was precleared (discarding unhomogenized particles and nuclei by a 600x g, 3-minute centrifugation followed by fractionation into crude cytosol (CC) and crude mitochondria (MC) by a 10-minute centrifugation at 10,000x g. The crude mitochondrial fraction obtained at this step contains fragments of the other cellular membranous organelles mostly ER or to a lesser extent, plasma membrane. In the next step, involving ultracentrifugation at 95,000x g for 30 minutes we separated pure mitochondria (MP) from mitochondria-associated membranes (MAM). Then, the well-purified mitochondria (MP) were pelleted at 6,300x g for 10 minutes. MAM fraction instead, was washed from Percoll® and pulled down by ultracentrifugation at 100,000x g for 1 h. Simultaneously, by ultracentrifugation of CC at 100,000x g for 1h, we obtained purified cytosolic fraction (CP) and microsomes (mostly ER membranes). The presented diagram summarizes the procedure. Those main steps were supplemented with additional centrifugations, aimed at the best possible purification of the processed fractions (more details in Wieckowski et. al 2009 [59]).

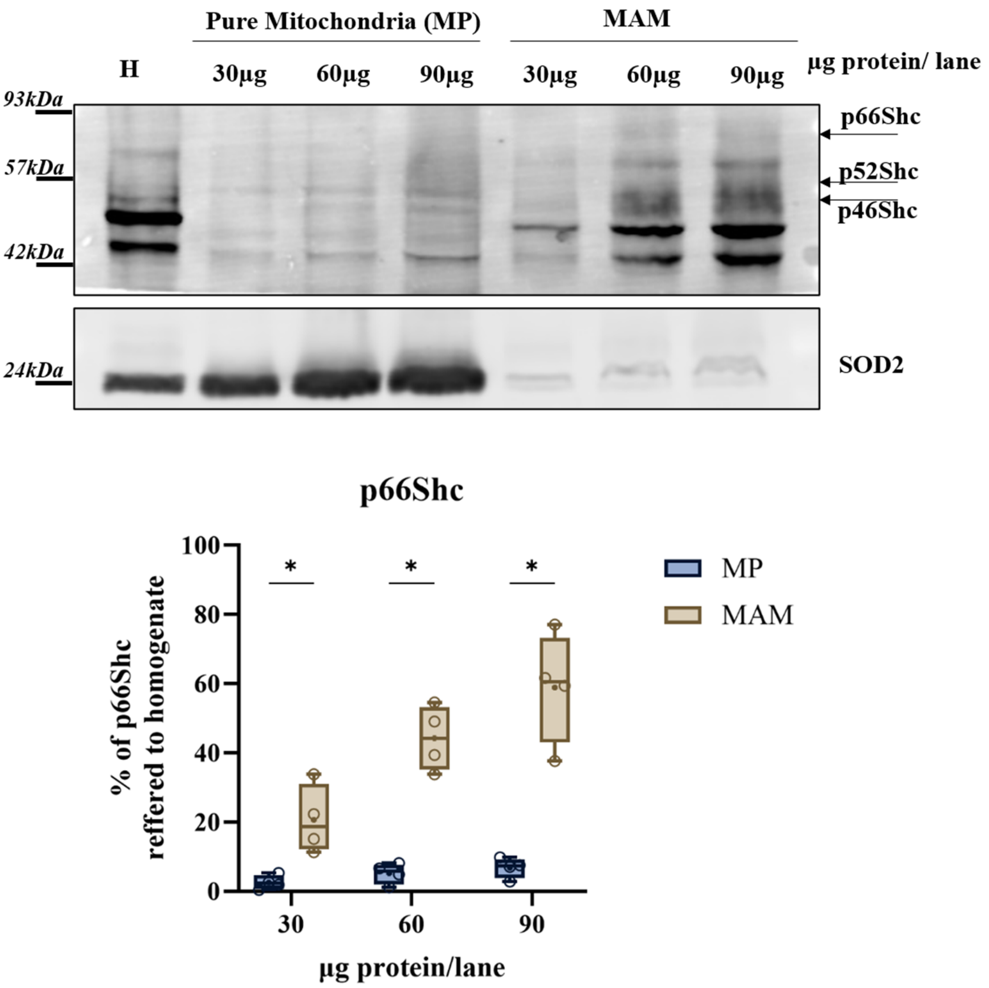

Figure S2: Verification of the specificity of p66Shc immunodetection in pure mitochondrial and MAM fractions.

Rising amounts of the total protein: 30 µg, 60 µg and 90 µg from the fractions' lysates of pure mitochondria (MP) and MAM were loaded onto an SDS-PAGE followed by western blot detection of p66Shc with specific anti-ShcA antibodies. The graph shows means from 4 replicates with SD. The means of p66Shc level in each sample were expressed as percentage, where the level of p66Shc in the homogenate sample (H) was 100%. SOD2 is shown as a positive control for mitochondrial protein loading. Statistical significance calculated with a t-test compares levels of p66Shc in MP and MAM for each amount of protein loaded (*t*-test MP vs MAM, *$p < 0.05$).

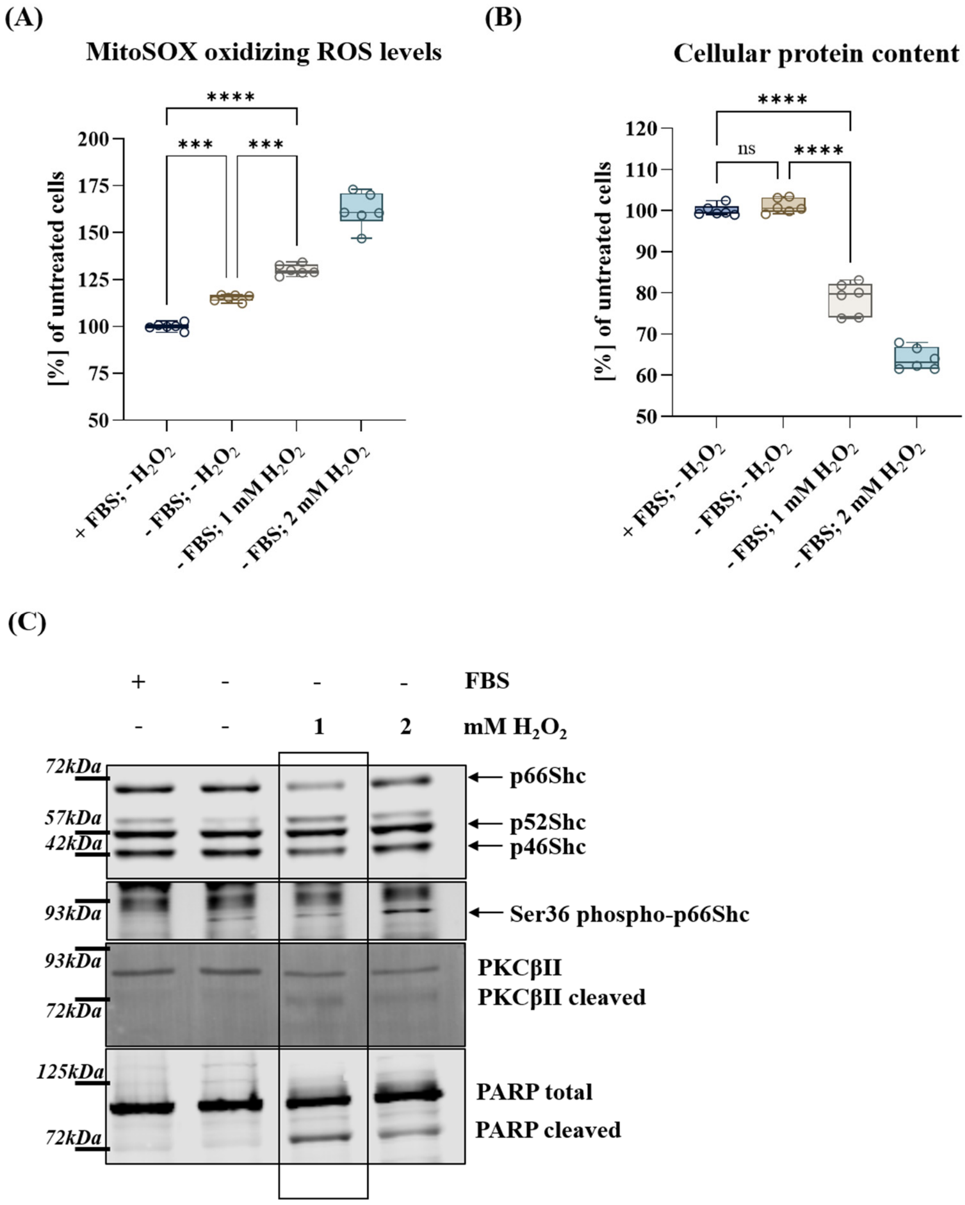

Figure S3: Parameters verifying oxidative stress induction in HepG2 cell culture.

(A) The level of MitoSOX™ fluorescent probe oxidizing reactive oxygen species (ROS) in HepG2 cell culture grown for 24 h in FBS-depleted media, FBS-depleted media with 1 mM H_2O_2 and FBS-depleted media with 2 mM H_2O_2 compared to untreated culture grown in normal, 10 % FBS containing media. **(B)** Cellular protein content in HepG2 cell culture cultured for 24 h in FBS-depleted media, FBS-depleted media with 1 mM H_2O_2 and 2 mM H_2O_2 assessed with sulforhodamine B (SRB) staining assay expressed as percentage of cellular protein content in untreated culture grown in 10 % FBS containing media. The graphs show means with SD from 1 representative biological replicate and 6 technical replicates. **(C)** Verification of the Serine – 36 phosphorylation pathway activation upon 24 h growth in FBS-depleted media, FBS-depleted media with 1 mM H_2O_2, and FBS-depleted media with 2 mM H_2O_2 in comparison to an untreated culture grown in normal, 10 % FBS containing media with western blot technique. PKCβII cleavage (one of its activation modes) manifests p66Shc - Ser36 phosphorylation activation, and PARP cleavage as a marker of apoptosis pathway activation. Box plots in panels (A) and (B) show median values with SD; ****p<0.0001, ***p<0.0005, ns – no significance calculated with one-way ANOVA with Tukey's method based multiple comparisons.

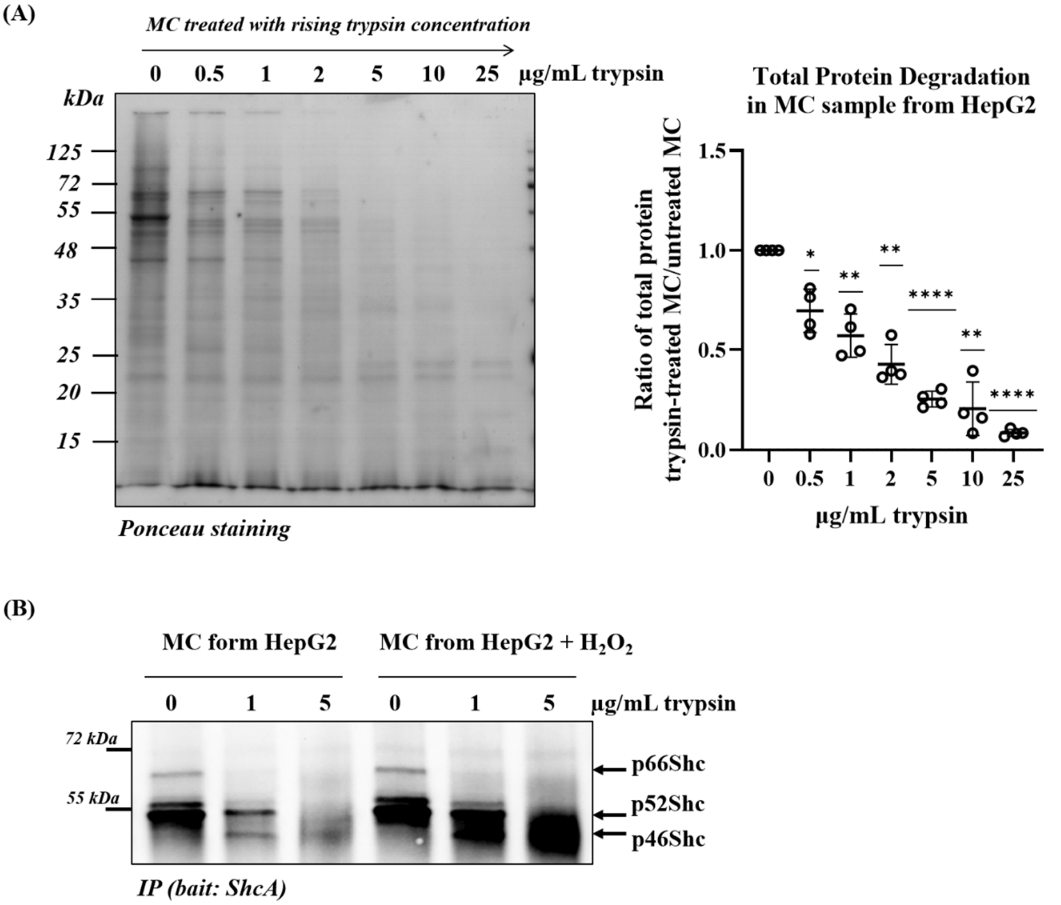

Figure S4: Verification of controlled digestion assay of proteins in crude mitochondria (MC) isolated from HepG2 cells.

(A) Total protein degradation pattern verifying the controlled digestion assay in MC isolated from HepG2 cell line. The membrane picture shows Ponceau staining performed before western blot detection of p66Shc and fraction markers presented in Figure 3A. 100 µg of protein from crude mitochondrial fraction was treated with rising trypsin concentrations from 0.5 µg/ml to 25 µg/ml trypsin for 15 minutes. The graph on the right shows the mean values of the total lane signal (total protein) quantified for 4 replicates with SDs. Statistical significance was calculated with one sample *t*-test (where value = 1 refers to the input – undigested MC sample), *p*-value (****<0.0001, **<0.005, *<0.05). **(B)** Western blot shows the level of p66Shc immunoprecipitated using specific anti-ShcA antibodies in the samples of crude mitochondria (MC) treated with 0.5 µg/ml and 5 µg/ml trypsin. H – homogenate.

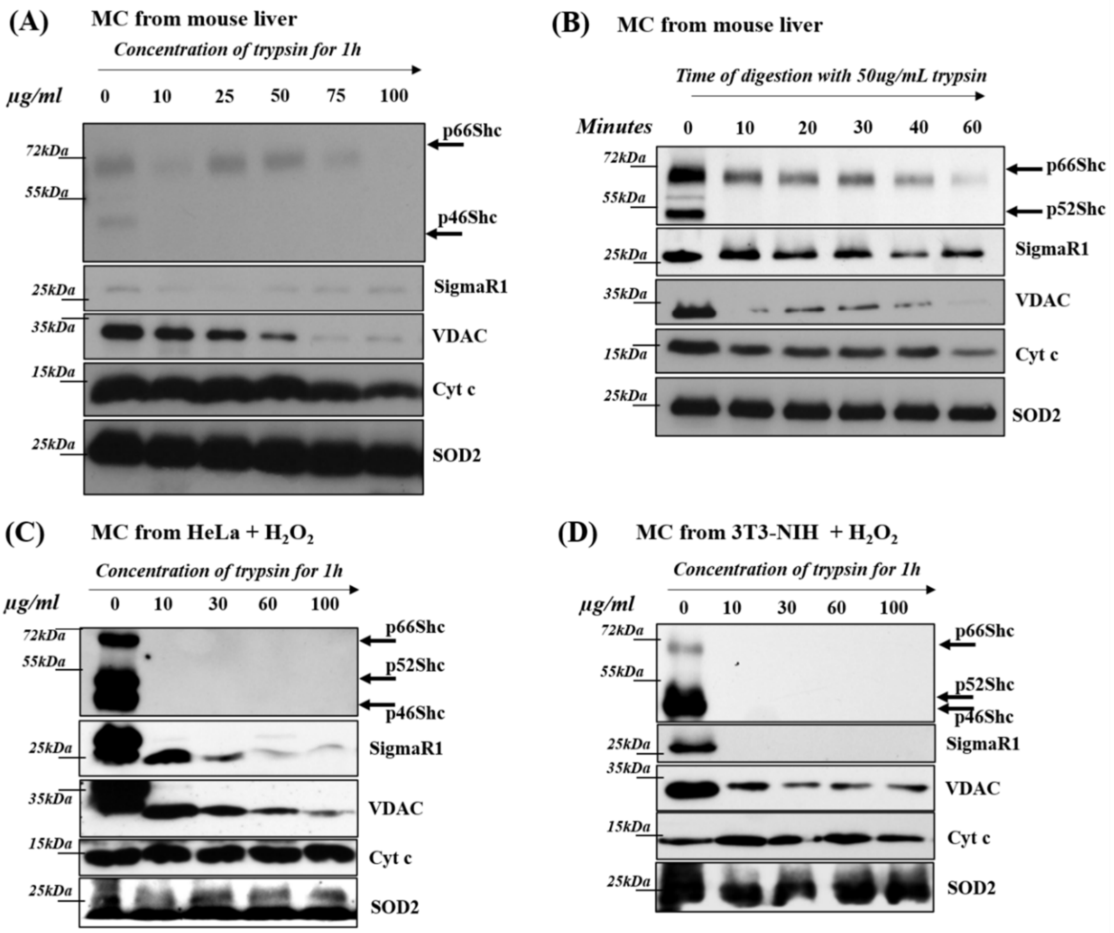

Figure S5: p66Shc level after trypsin digestion of MC fraction from mice liver, and from HeLa and 3T3 cell lines.

ShcA proteins digestion pattern in MC treated with **(A)** rising trypsin concentrations: 10, 25, 50, 75, 100 µg/ml for 1 h, and **(B)** time-dependent manner 10, 20, 30, 40, 60 minutes with 50 µg of trypsin; **(C)** The level of ShcA proteins after digestion with trypsin (range of concentrations: 10 µg/ml - 100 µg/ml) of 100 µg of MC isolated from the HeLa cell line treated for 1h with 500 µM H_2O_2 in phosphate-buffered saline (PBS) containing Ca^{2+} and Mg^{2+} ions and supplemented with 25 mM glucose, and **(D)** from 3T3 - NIH fibroblasts treated for 30 minutes with 500 µM H_2O_2 in phosphate-buffered saline (PBS) containing Ca^{2+} and Mg^{2+} ions and supplemented with 25 mM glucose, followed by the detection of fractions markers: SigmaR1 or ACSL4 for MAM, VDAC for outer mitochondrial membrane (OMM), Cyt c for intermembrane space (IMS) and SOD2 for mitochondrial matrix (MM) in the residual samples after digestion. Single trials.

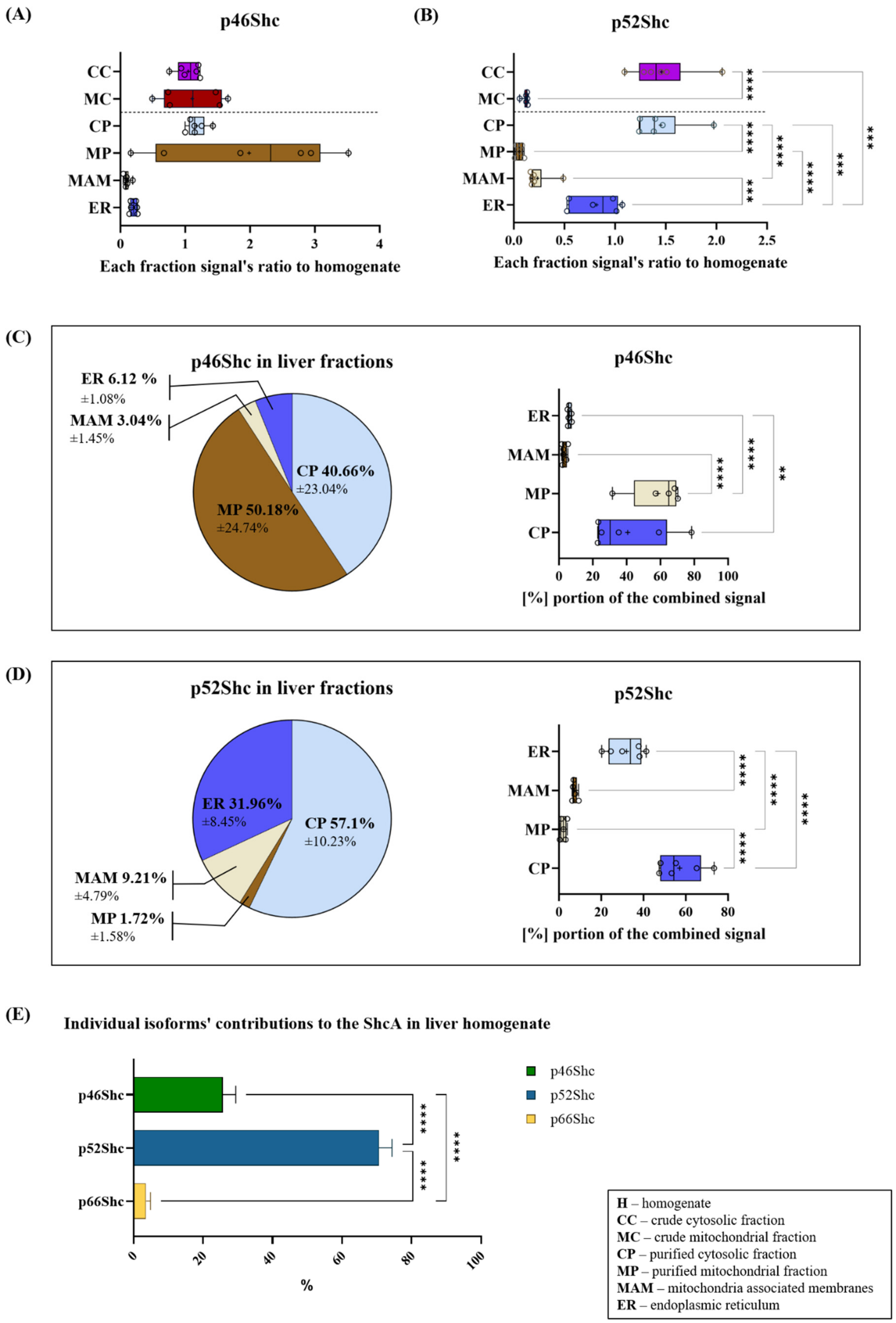

Figure S6: p46Shc and p52Shc distribution in cellular fractions isolated from mouse liver.

The levels of p46Shc **(A)** and p52Shc **(B)** are expressed as a ratio of western blot signal (shown in Figure 1B) in separated fractions to the level in homogenate isolated from mice livers. Box plots show the medians (lines), means are indicated with (+); $n = 6$; Statistical significance evaluated with ordinary one-way ANOVA with Tukey's method-based multiple comparisons (****$p < 0.0001$, ***$p < 0.0005$); The percentage contribution of p46Shc **(C)** and p52Shc **(D)** in each of pure fractions in the total (100%) of the respective protein content calculated as a sum of values from MP, MAM, CP, and ER fractions (pie chart shows mean percentage representation of p46Shc (C) and p52Shc (D) in each fraction and box plot shows the mean with SD); $n = 6$; Statistical significance evaluated with ordinary one-way ANOVA with Tukey's method based multiple comparisons (****$p < 0.0001$, **$p < 0.005$); **(E)** The contribution of each ShcA isoform (p46Shc, p52Shc, p66Shc) in the total ShcA western blot signal in total homogenate isolated from mice livers; Bar chart shows mean values with SD; $n = 6$; Statistical significance evaluated with ordinary one-way ANOVA with Tukey's method based multiple comparisons (****$p < 0.0001$). The lower right corner displays a legend for the abbreviations for fraction names.

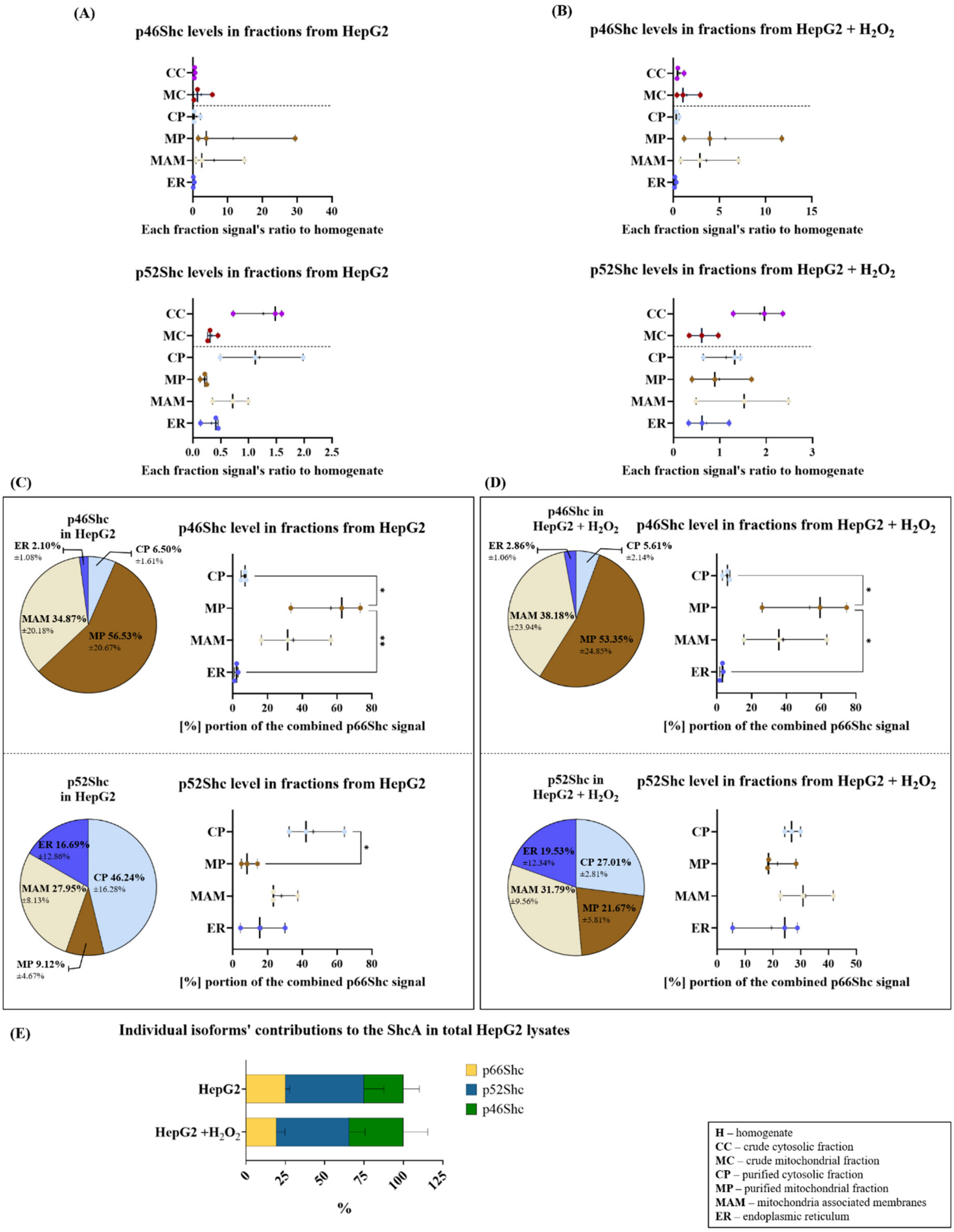

Figure S7: p46Shc and p52Shc distribution in cellular fractions isolated from HepG2 cells.

The levels of p46Shc and p52Shc in fractions isolated from control HepG2 cells **(A)** and from H_2O_2-treated HepG2 cells **(B)** expressed as ratios of western blot signals (shown in Figure 2B and 2C respectively) in separated fractions to the level in homogenate. Plots show the medians (lines), means are indicated with (+); *n* = 3; Statistical significance evaluated with ordinary one-way ANOVA with Tukey's method-based multiple comparisons (*****p* < 0.0001, ****p* < 0.0005); The percentage contribution of p46Shc and p52Shc in each of pure fractions isolated from control HepG2 cells **(C)** and from H_2O_2-treated HepG2 cells **(D)** in the total (100%) of the respective protein

content calculated as a sum of values from MP, MAM, CP, and ER fractions (pie charts show mean percentage representation of p46Shc p52Shc in each fraction and plots show the means with SD); $n = 3$; Statistical significance evaluated with ordinary one-way ANOVA with Tukey's method based multiple comparisons (**$p < 0.005$; *$p < 0.05$)); **(E)** The contribution of each ShcA isoform (p46Shc, p52Shc, p66Shc) expressed in percentage of the total ShcA western blot signal in total homogenate isolated from control HepG2 cells and from H_2O_2-treated HepG2 cells; Bar chart shows mean values with SD; $n = 3$. The lower right corner displays a legend for the abbreviations for fraction names.
